# Supplementary material for: Aeromonas spp. Prevalence, Virulence, and Antimicrobial Resistance in an Ex Situ Program for Threatened Freshwater Fish—A Pilot Study with Protective Measures
Source: Animals (Basel). 2022 Feb 11;12(4):436. doi: 10.3390/ani12040436 (PMC8868083; doi:10.3390/ani12040436)
Supplement: Supplementary file 1 [file animals-12-00436-s001.zip › 2.23 animals-1542936-supplementary/Supplementary Figure S2.pptx]

## Slide 1
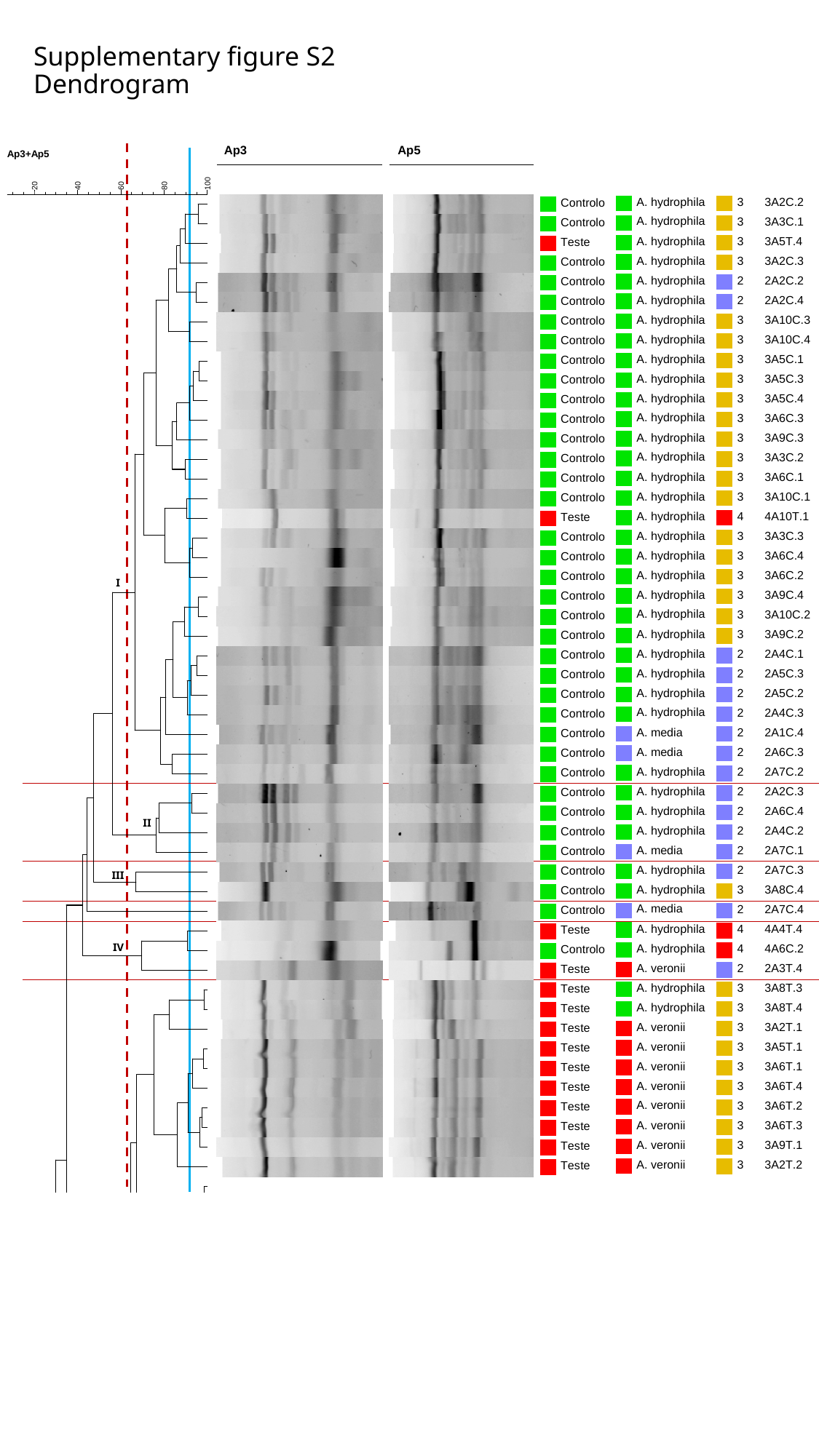

# Supplementary figure S2 Dendrogram
Ap3
Ap5
I
II
III
IV

## Slide 2
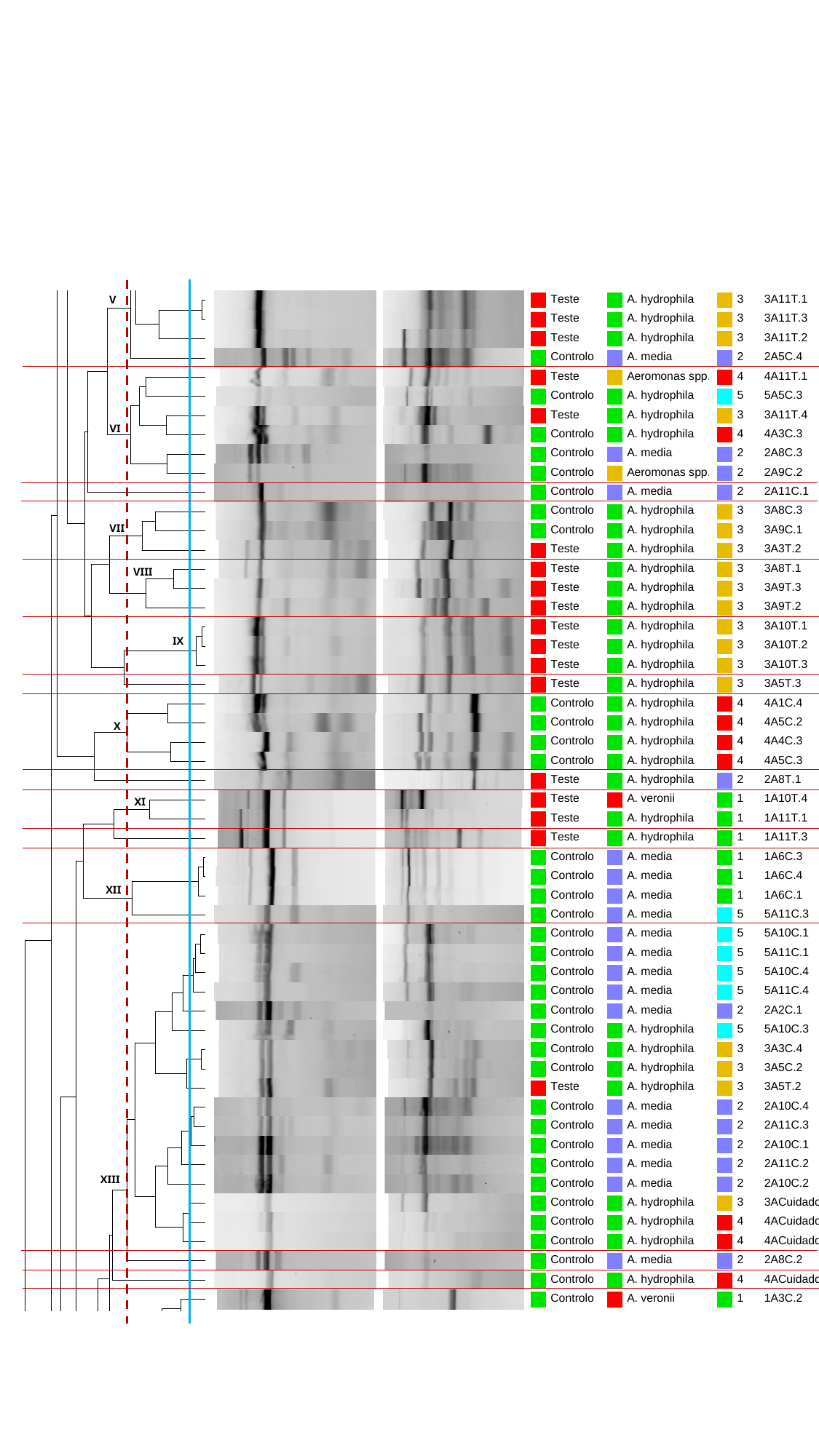

V
VI
VII
VIII
IX
X
XI
XII
XIII

## Slide 3
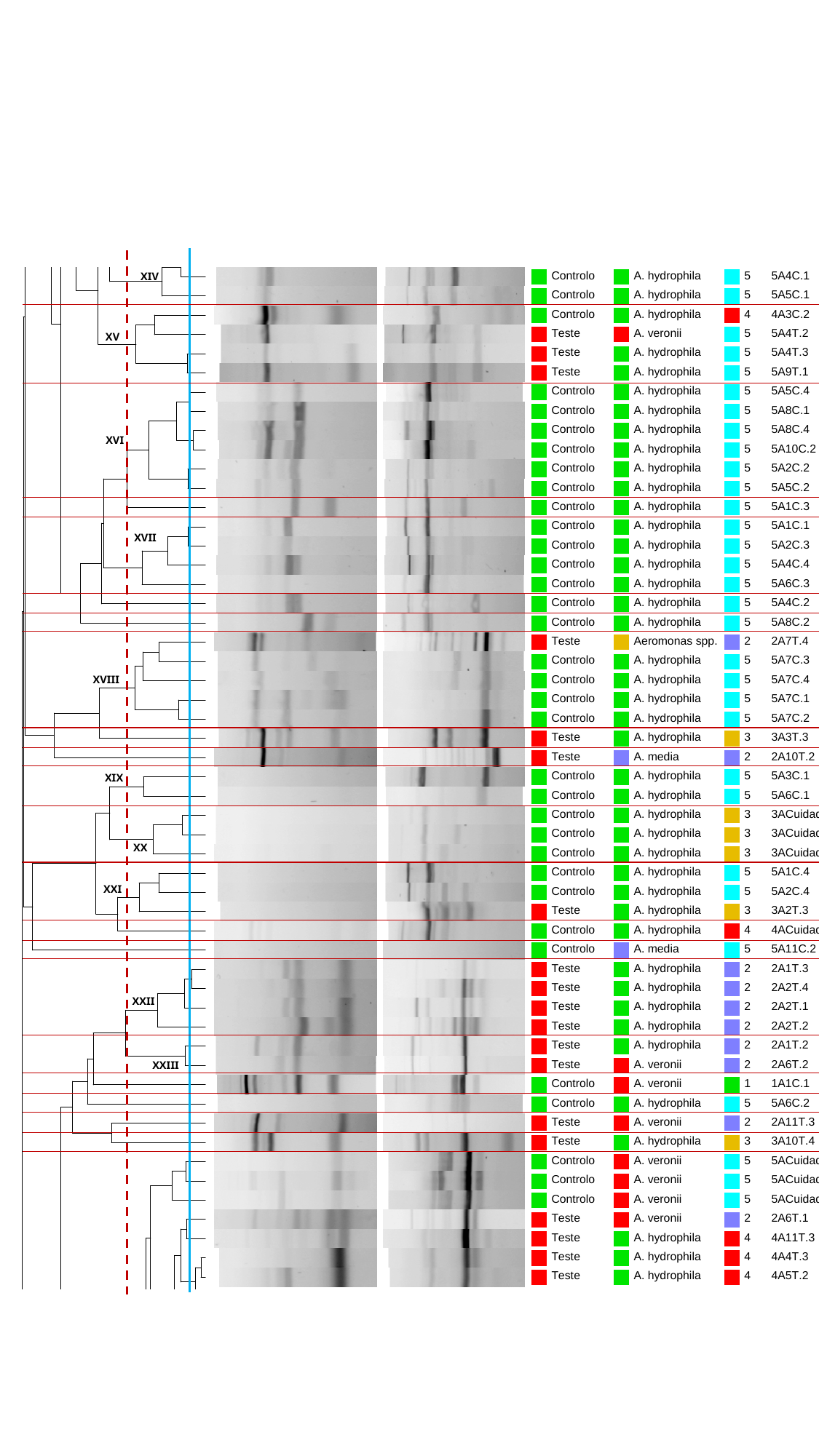

XIV
XV
XVI
XVII
XVIII
XIX
XX
XXI
XXII
XXIII

## Slide 4
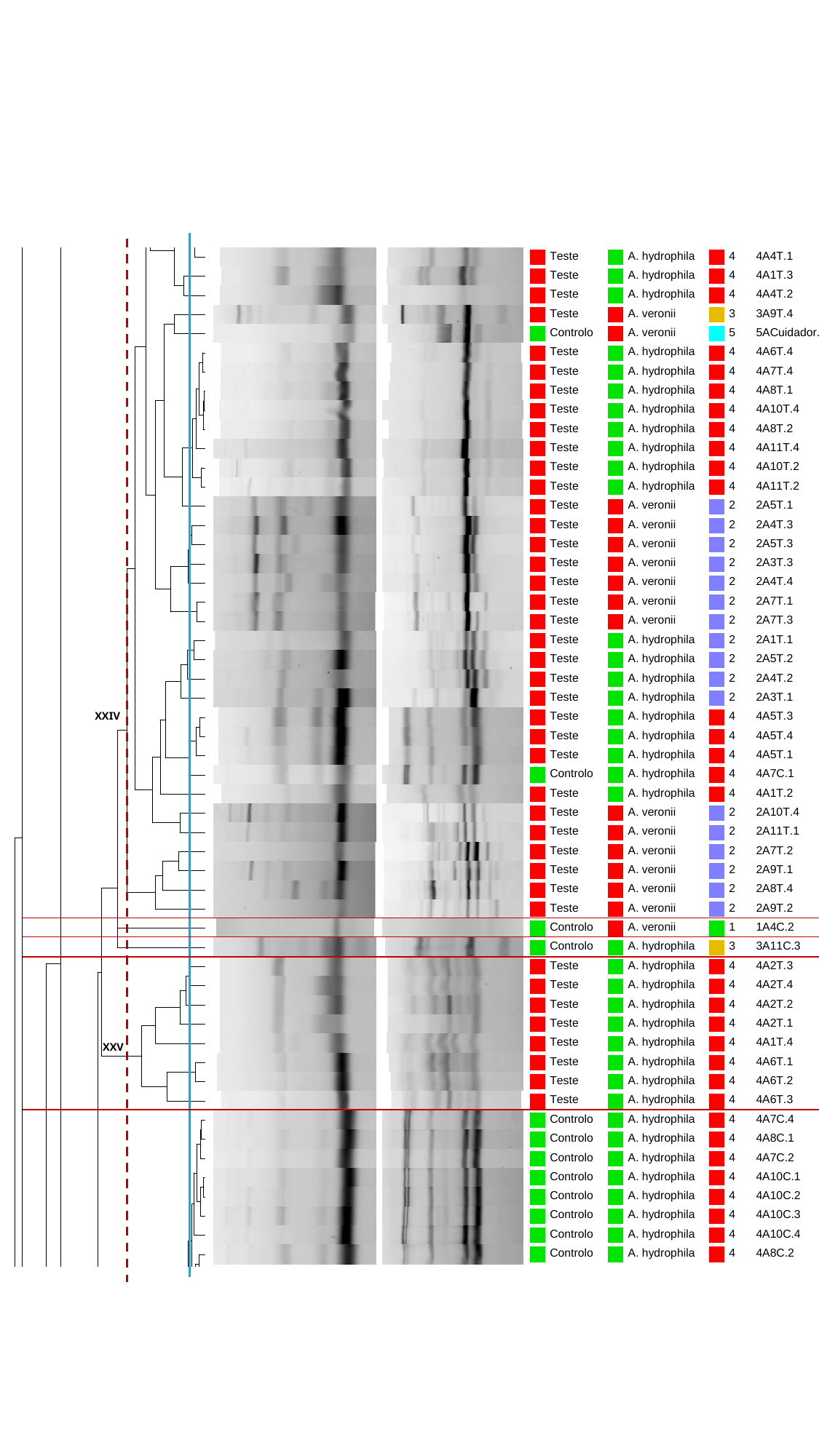

XXIV
XXV

## Slide 5
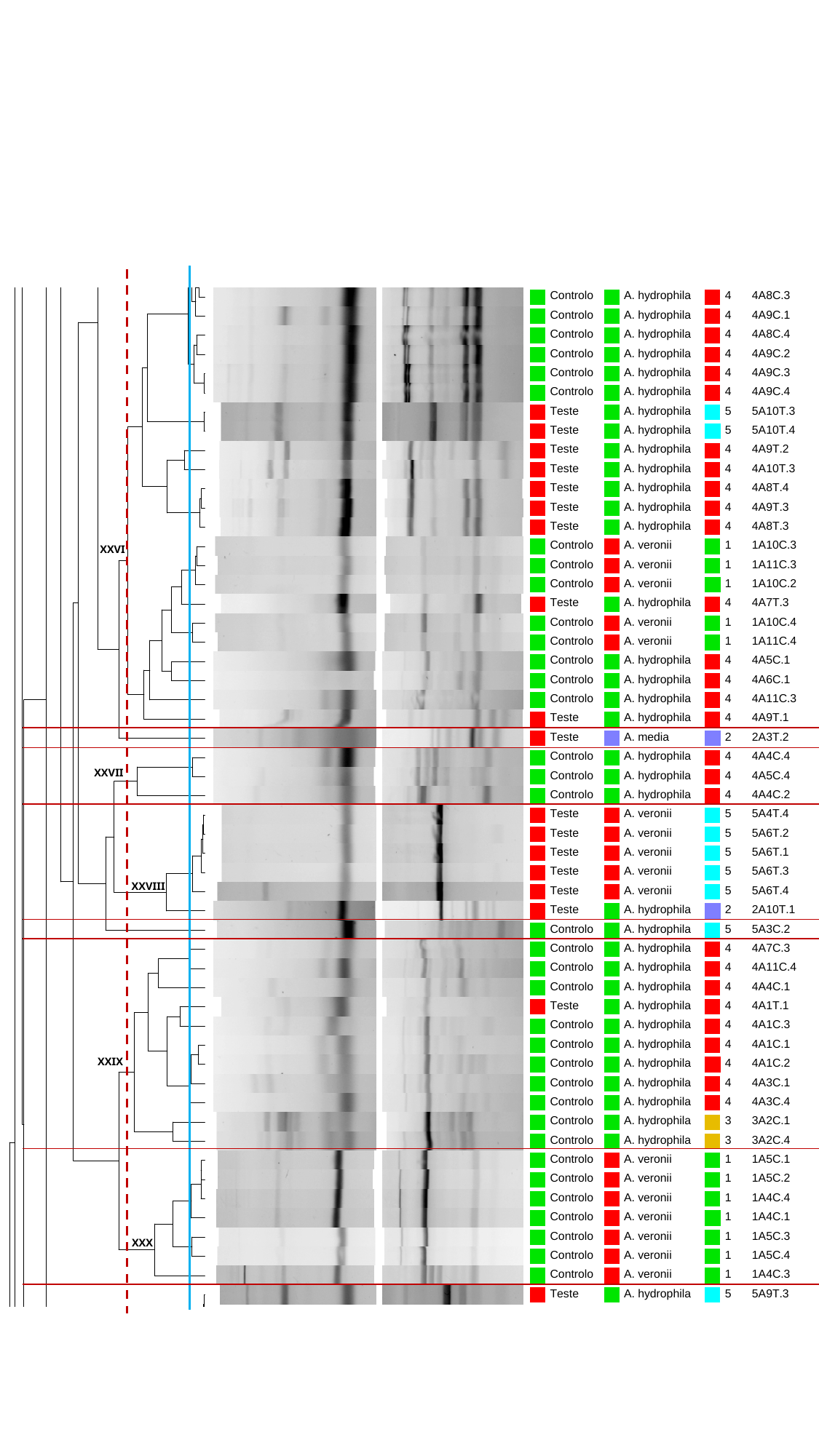

XXVI
XXVII
XXVIII
XXIX
XXX

## Slide 6
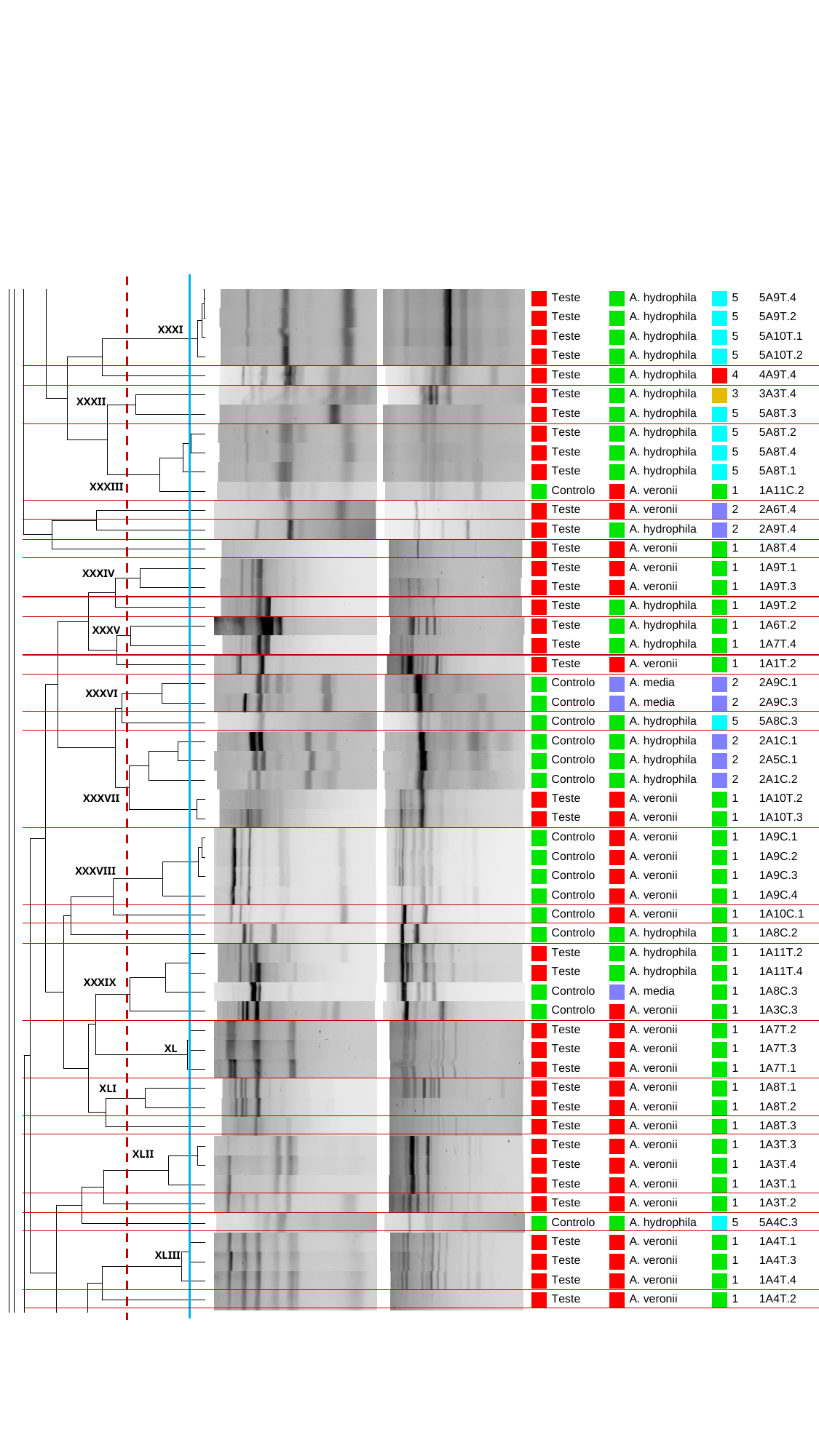

XXXI
XXXII
XXXIII
XXXIV
XXXV
XXXVI
XXXVII
XXXVIII
XXXIX
XL
XLI
XLII
XLIII

## Slide 7
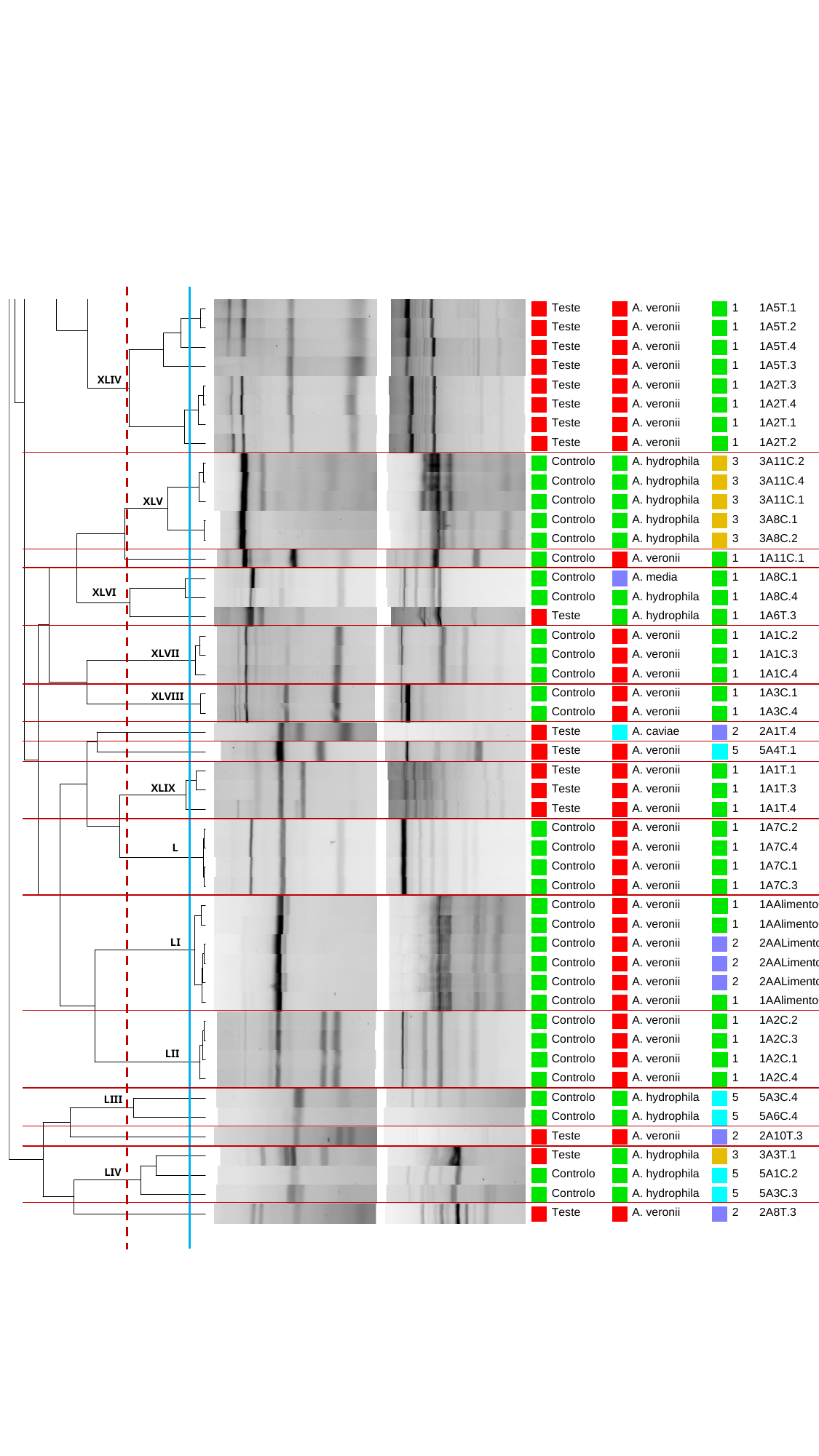

XLIV
XLV
XLVI
XLVII
XLVIII
XLIX
L
LI
LII
LIII
LIV
