# Supplementary material for: Aeromonas spp. Prevalence, Virulence, and Antimicrobial Resistance in an Ex Situ Program for Threatened Freshwater Fish—A Pilot Study with Protective Measures
Source: Animals (Basel). 2022 Feb 11;12(4):436. doi: 10.3390/ani12040436 (PMC8868083; doi:10.3390/ani12040436)
Supplement: Supplementary file 1 [file animals-12-00436-s001.zip › 2.23 animals-1542936-supplementary/Supplementary Figure S3 & S4.pptx]

## Slide 1
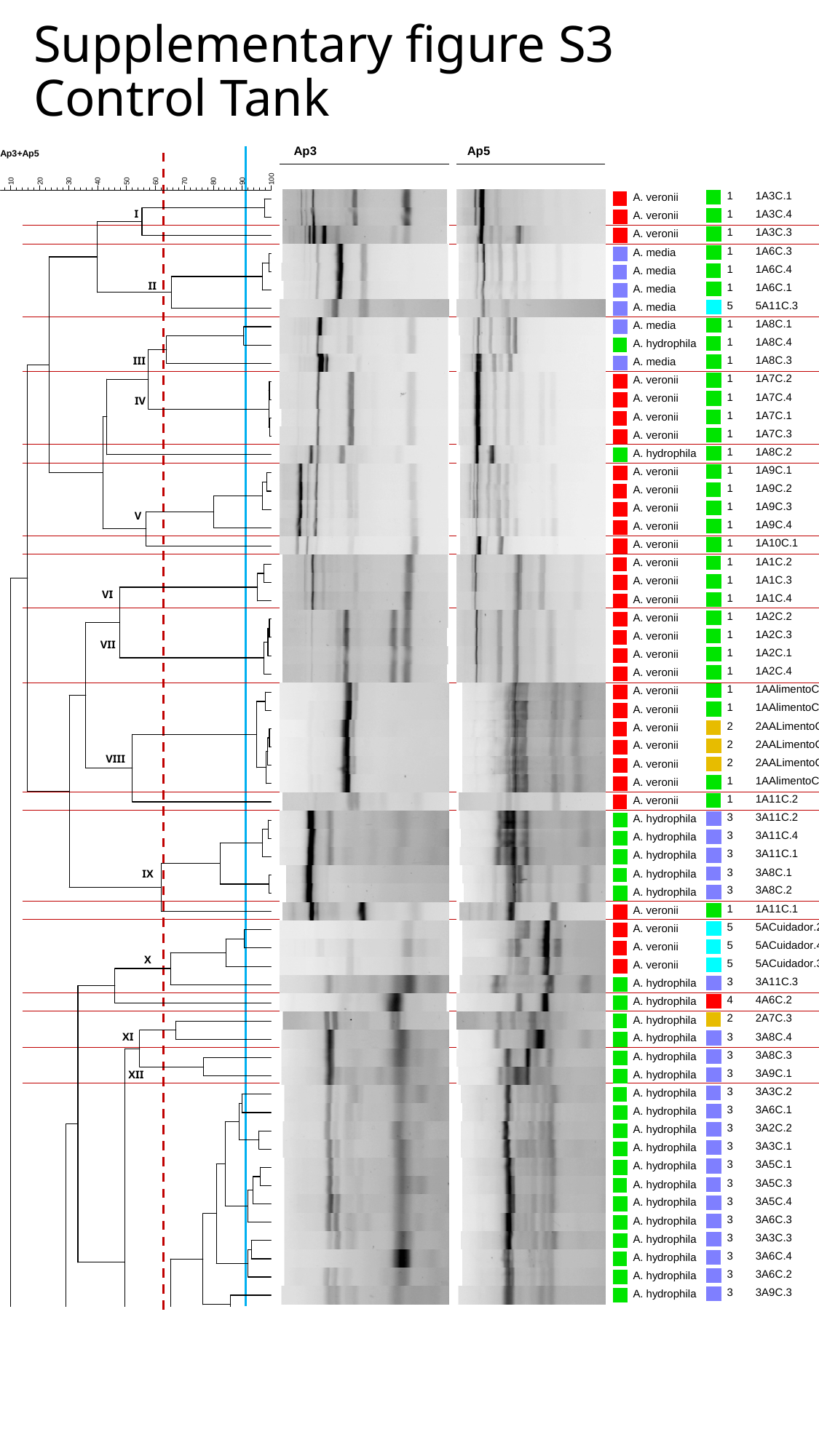

# Supplementary figure S3 Control Tank
Ap3
Ap5
I
II
III
IV
V
VI
VII
VIII
IX
X
XI
XII

## Slide 2
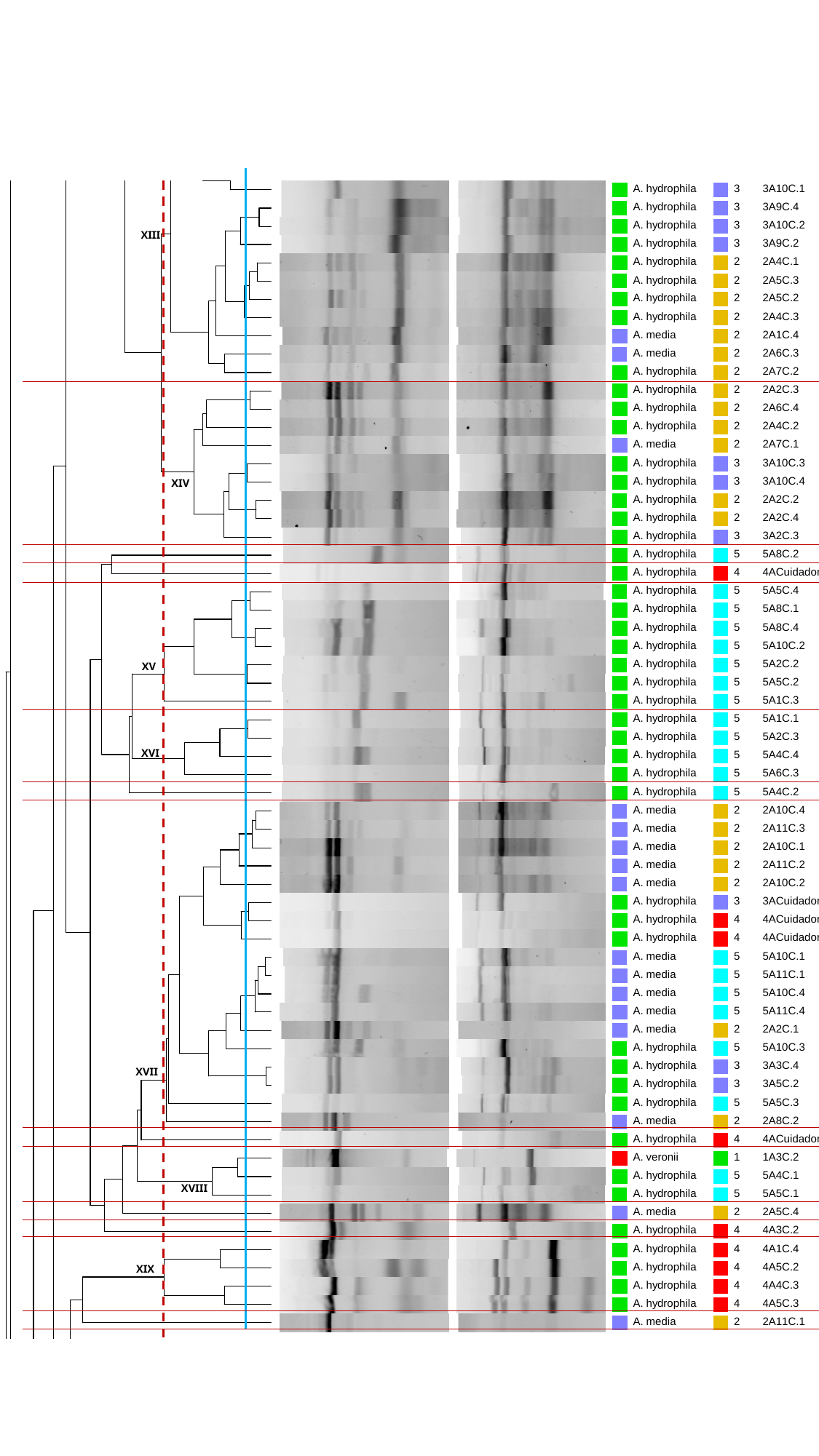

XIII
XIV
XV
XVI
XVII
XVIII
XIX

## Slide 3
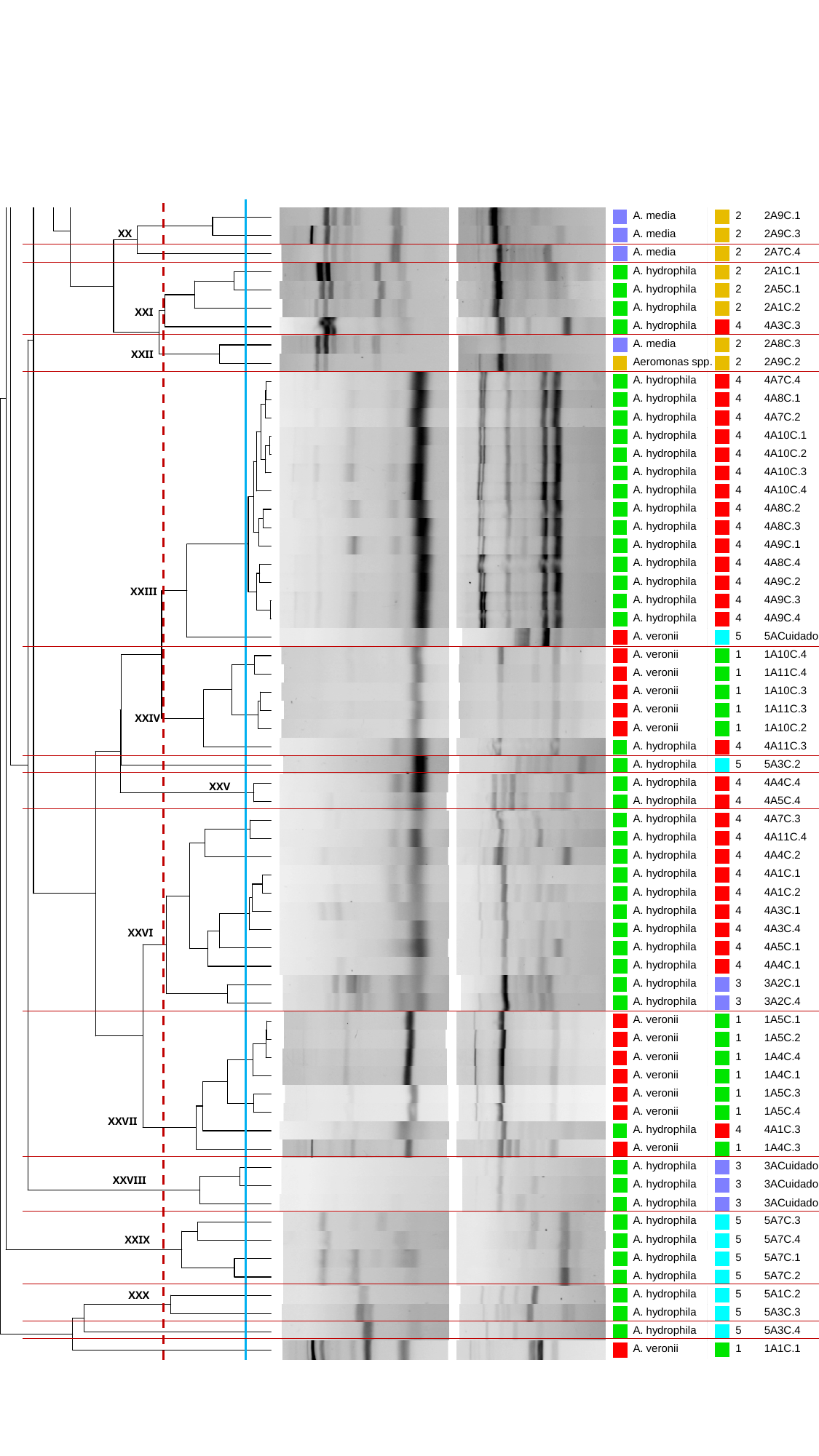

XX
XXI
XXII
XXIII
XXIV
XXV
XXVI
XXVII
XXVIII
XXIX
XXX

## Slide 4
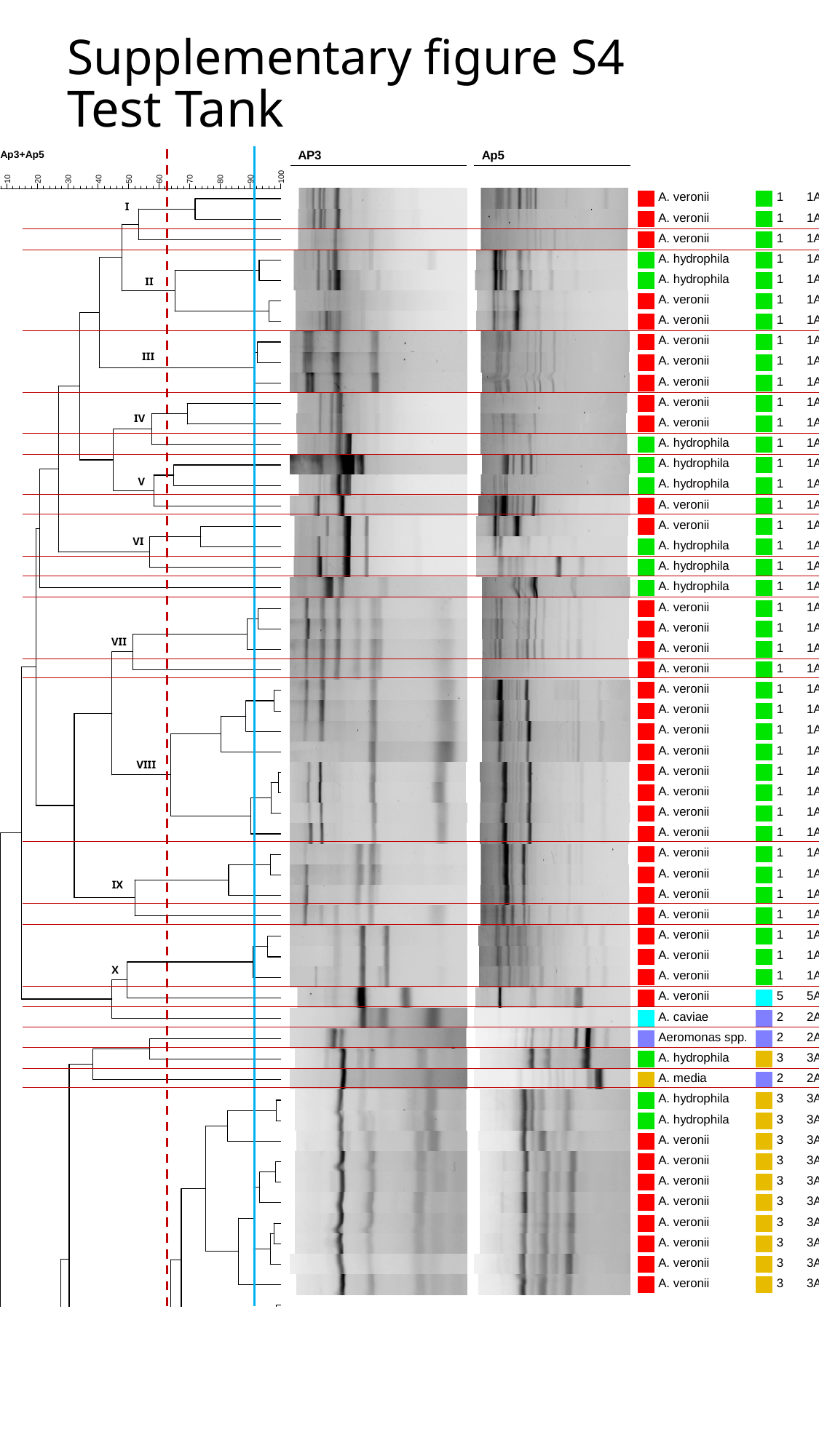

Supplementary figure S4
# Test Tank
I
II
III
IV
V
VI
VII
VIII
IX
X

## Slide 5
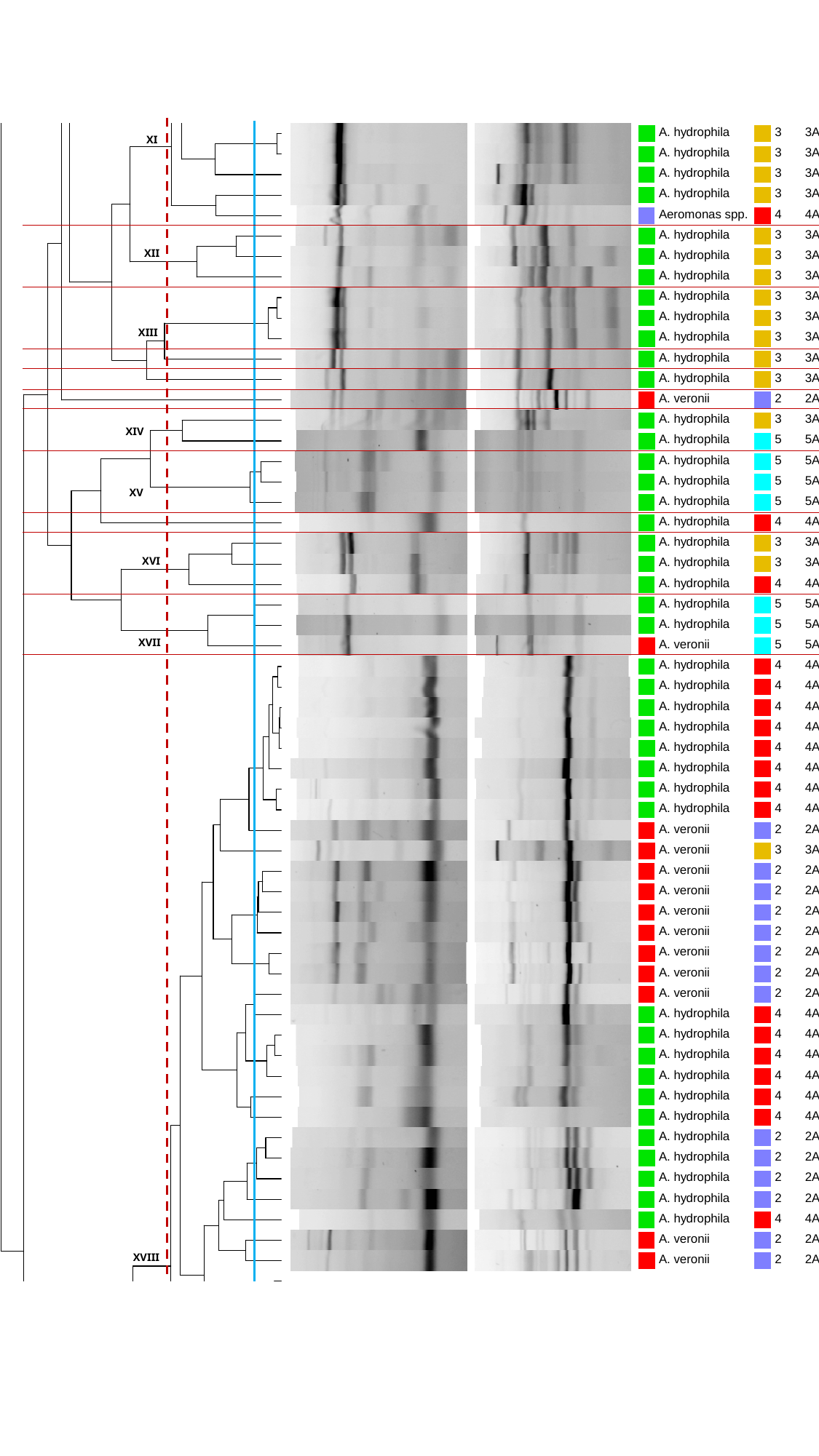

XI
XII
XIII
XIV
XV
XVI
XVII
XVIII

## Slide 6
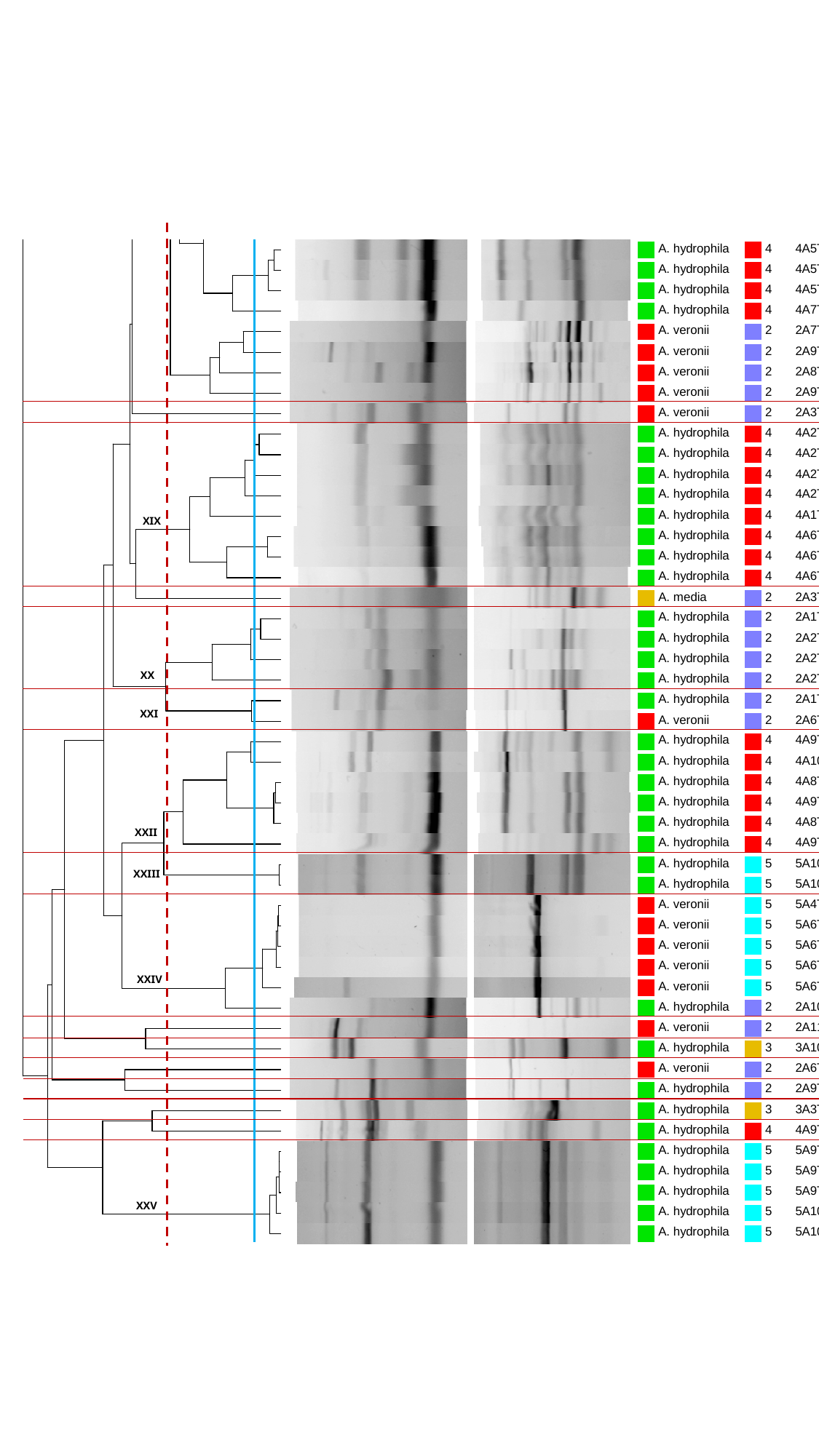

XIX
XX
XXI
XXII
XXIII
XXIV
XXV
