# Supplementary material for: Aeromonas spp. Prevalence, Virulence, and Antimicrobial Resistance in an Ex Situ Program for Threatened Freshwater Fish—A Pilot Study with Protective Measures
Source: Animals (Basel). 2022 Feb 11;12(4):436. doi: 10.3390/ani12040436 (PMC8868083; doi:10.3390/ani12040436)
Supplement: Supplementary file 1 [file animals-12-00436-s001.zip › 2.23 animals-1542936-supplementary/Supplementary Figure S5.pptx]

## Slide 1
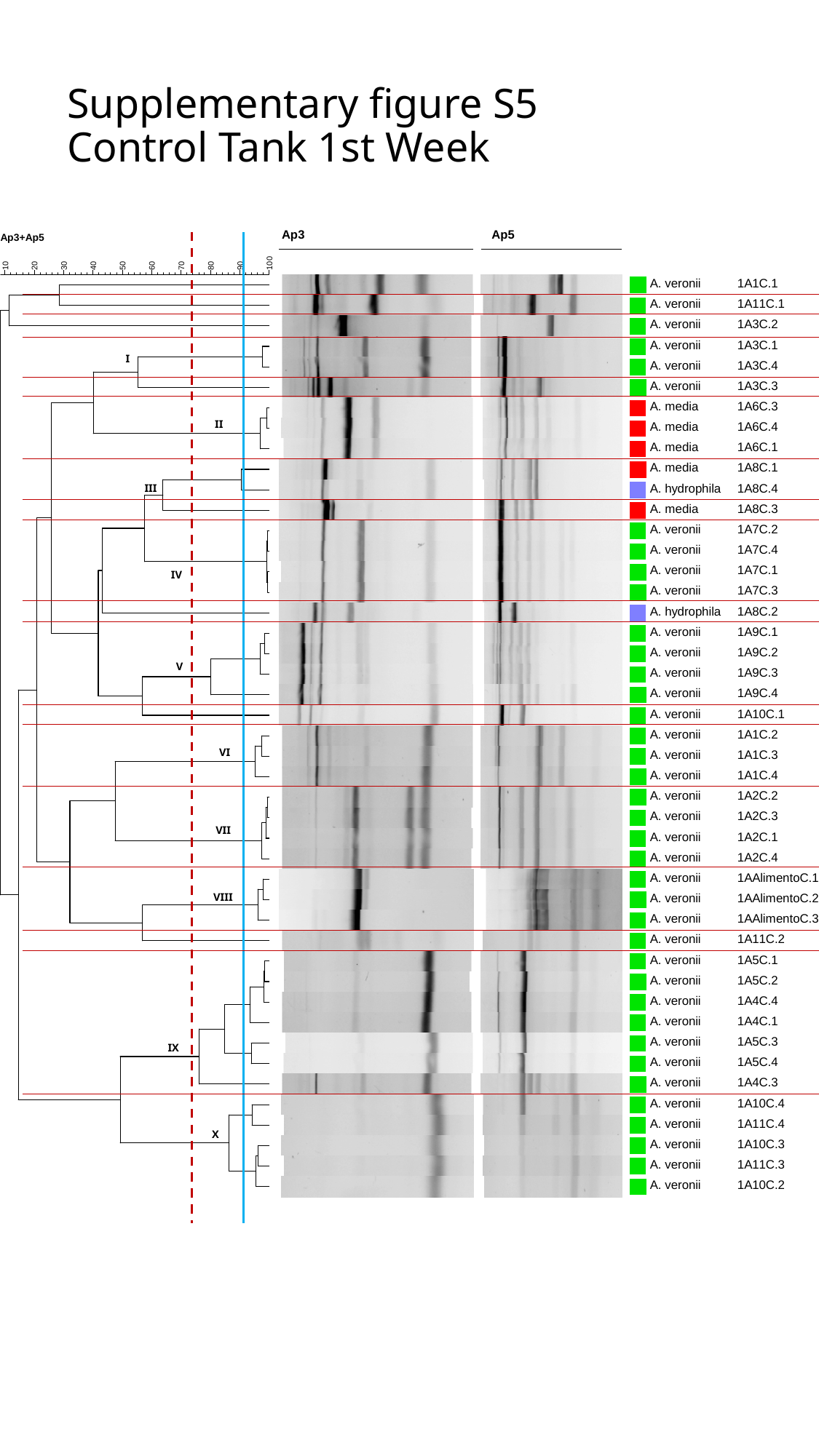

# Supplementary figure S5 Control Tank 1st Week
Ap3
Ap5
I
II
III
IV
V
VI
VII
VIII
IX
X

## Slide 2
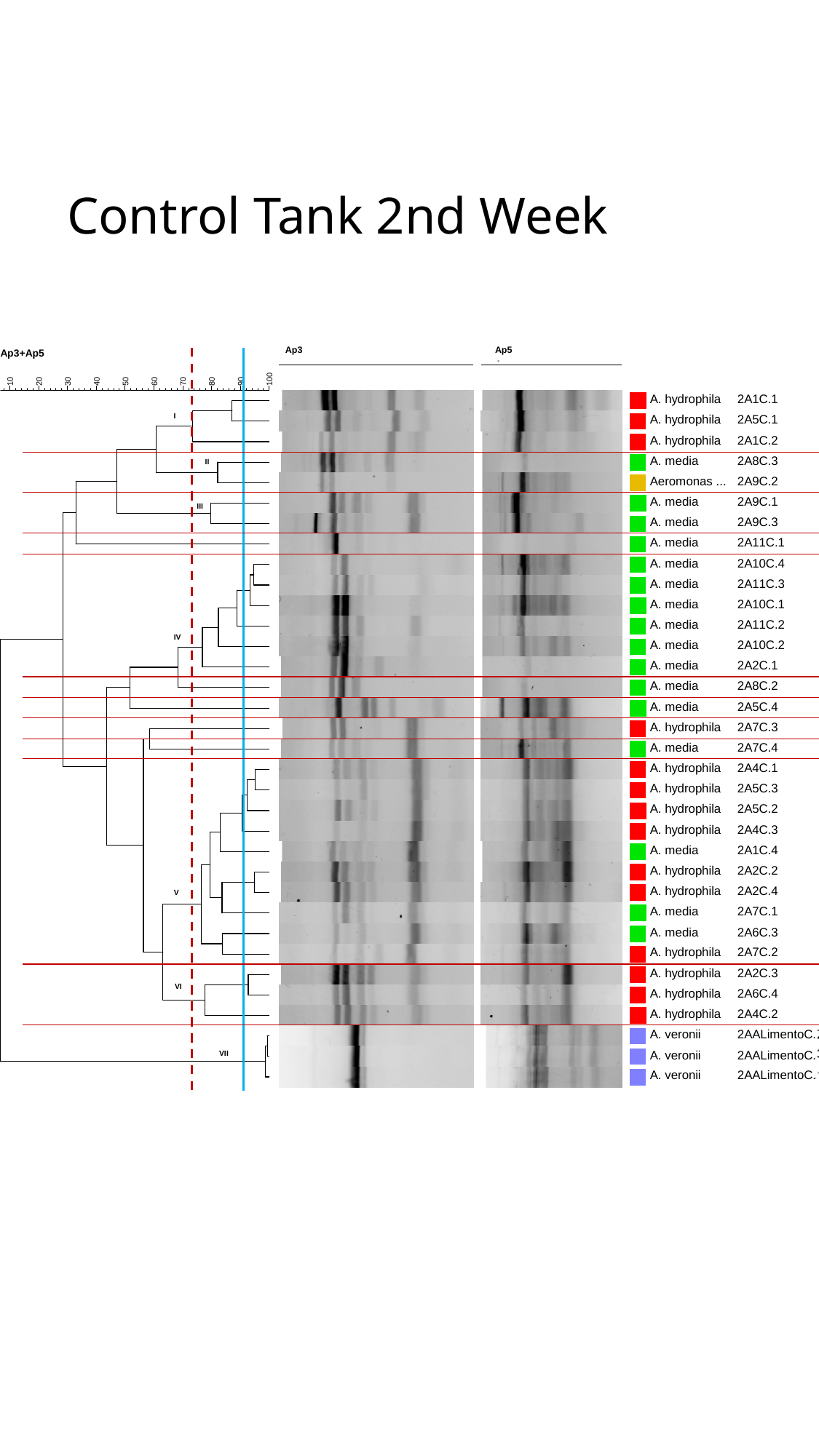

# Control Tank 2nd Week
Ap3
Ap5
I
II
III
IV
V
VI
VII

## Slide 3
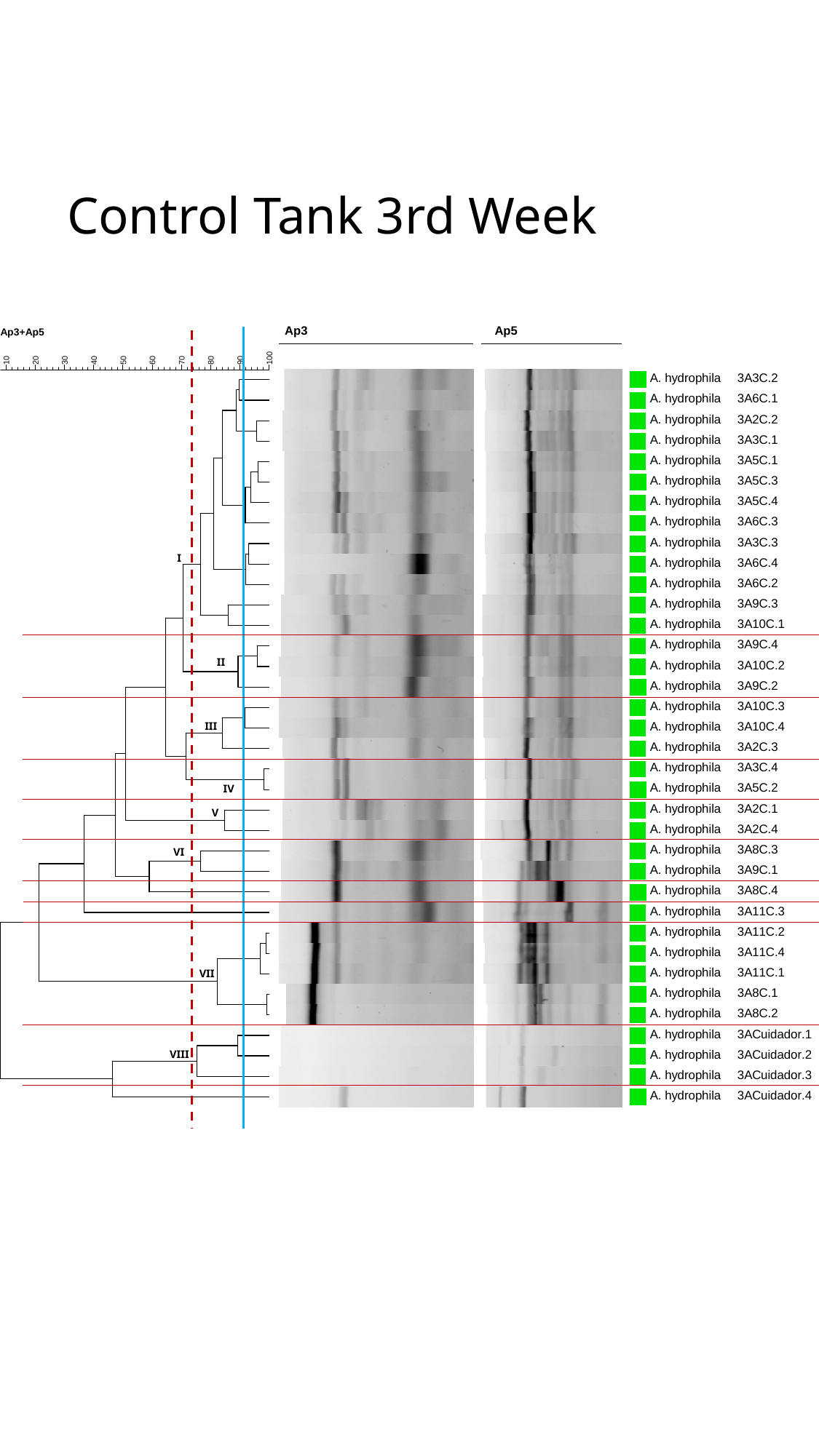

# Control Tank 3rd Week
Ap3
Ap5
I
II
III
IV
V
VI
VII
VIII

## Slide 4
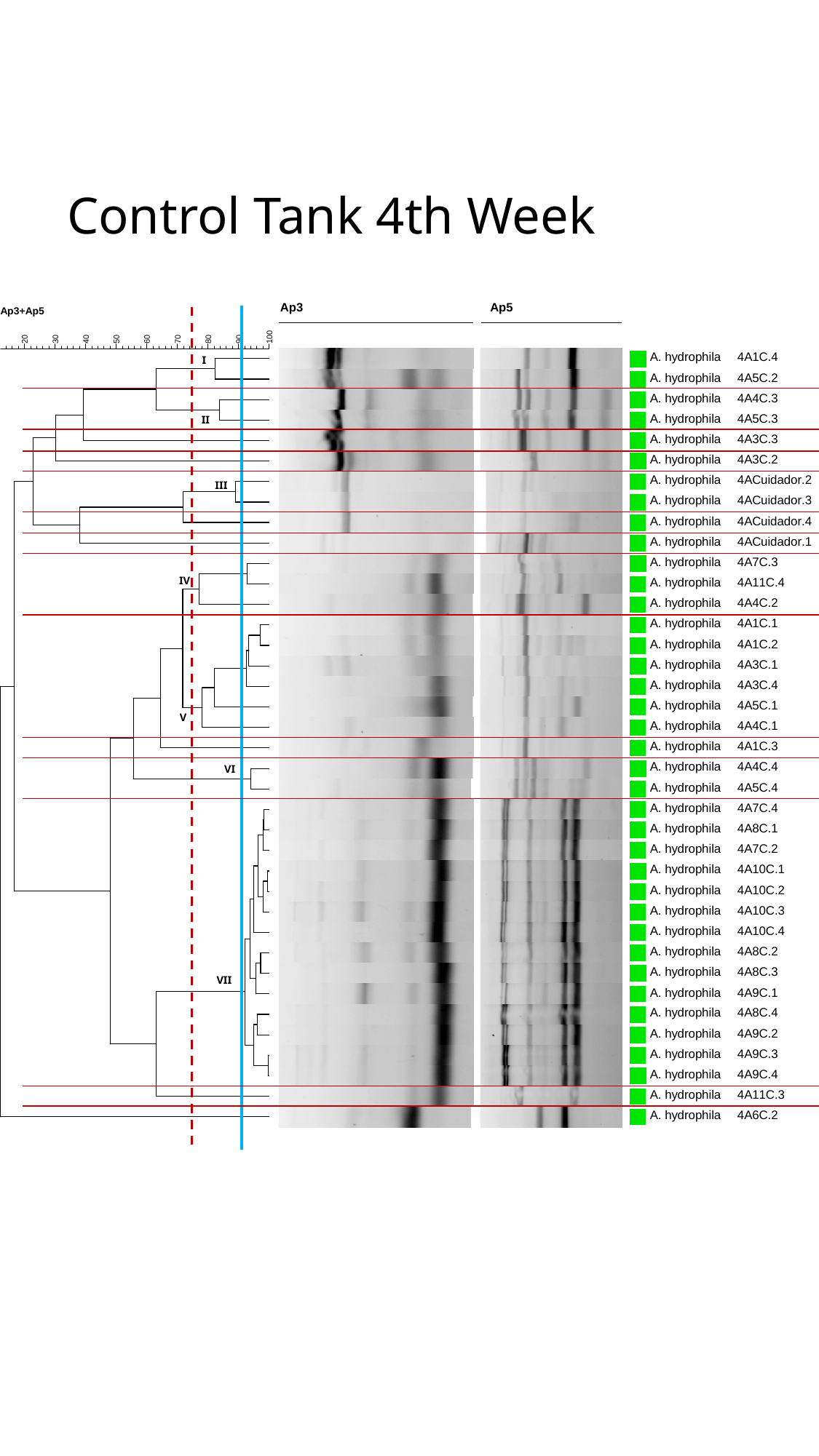

# Control Tank 4th Week
Ap3
Ap5
I
II
III
IV
V
VI
VII

## Slide 5
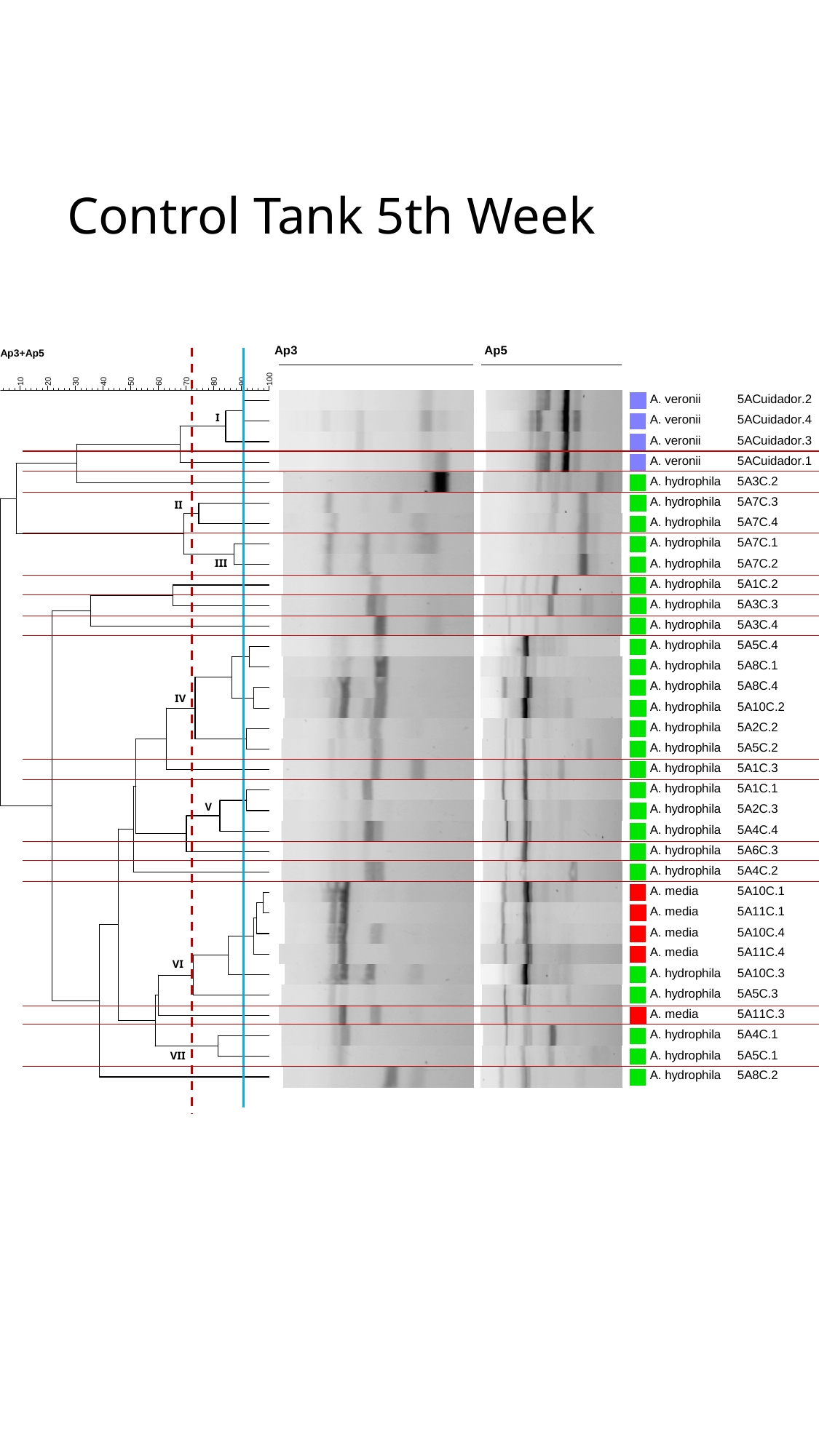

# Control Tank 5th Week
Ap3
Ap5
I
II
III
IV
V
VI
VII

## Slide 6
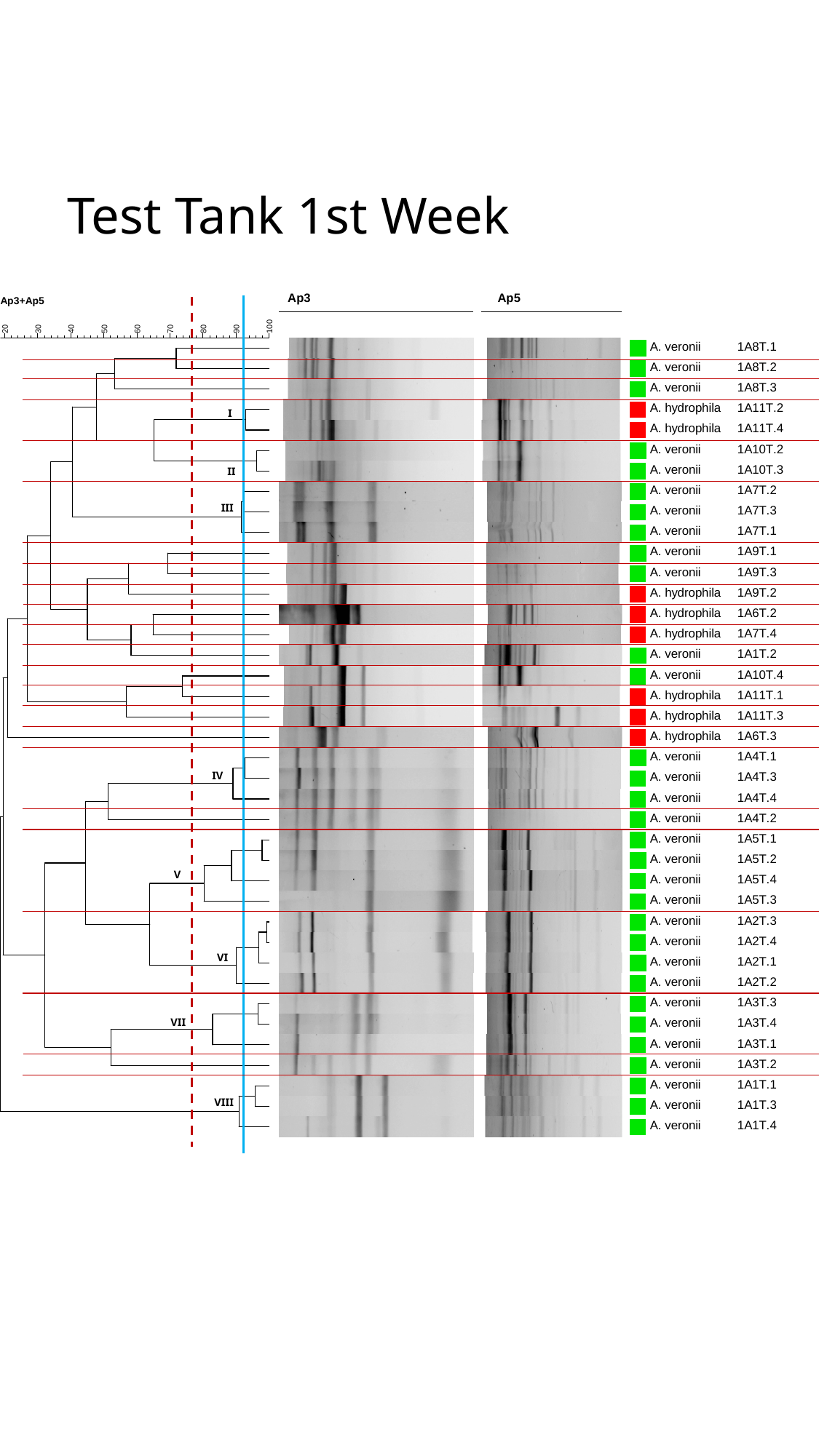

# Test Tank 1st Week
Ap3
Ap5
I
II
III
IV
V
VI
VII
VIII

## Slide 7
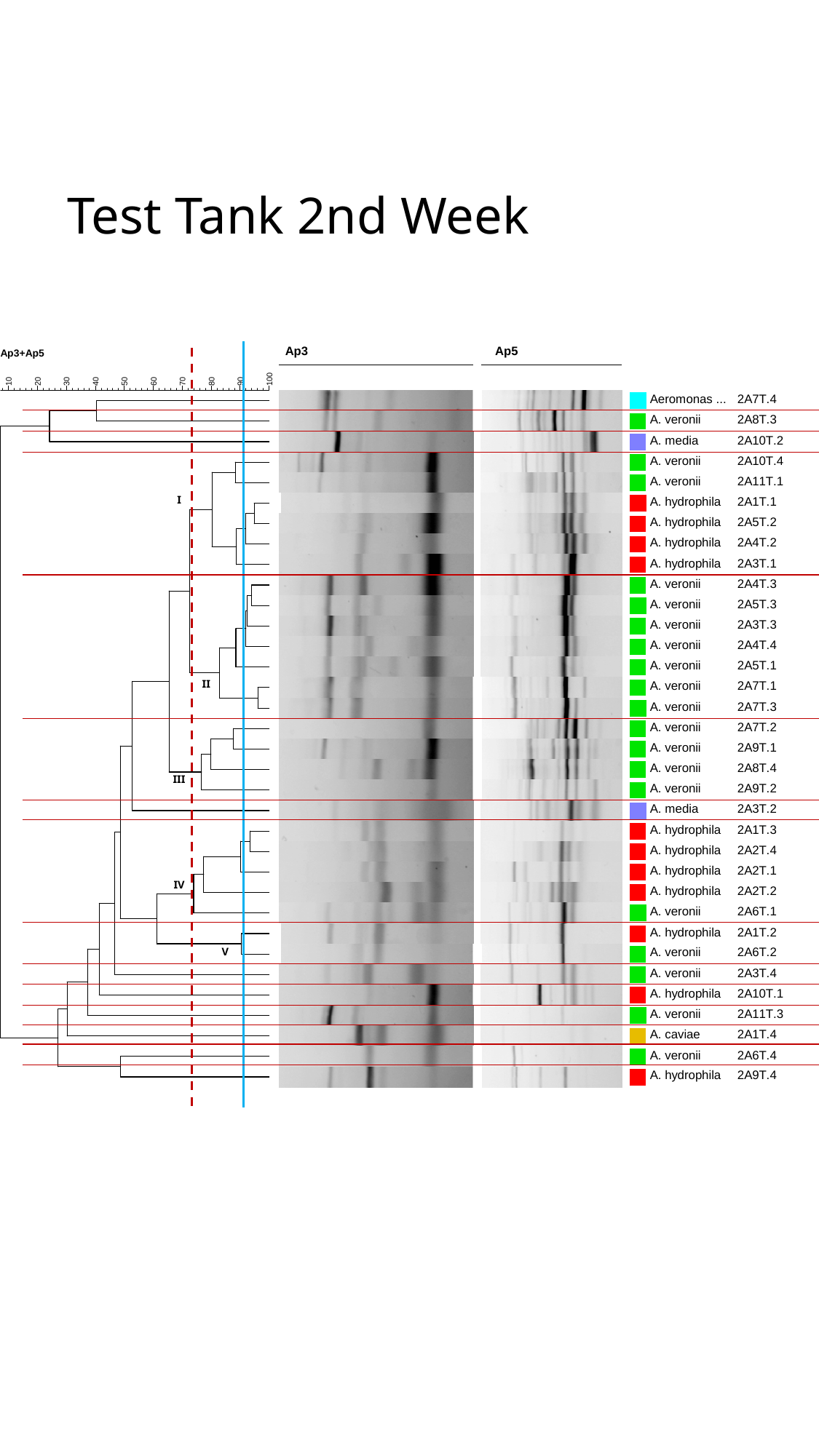

# Test Tank 2nd Week
Ap3
Ap5
I
II
III
IV
V

## Slide 8
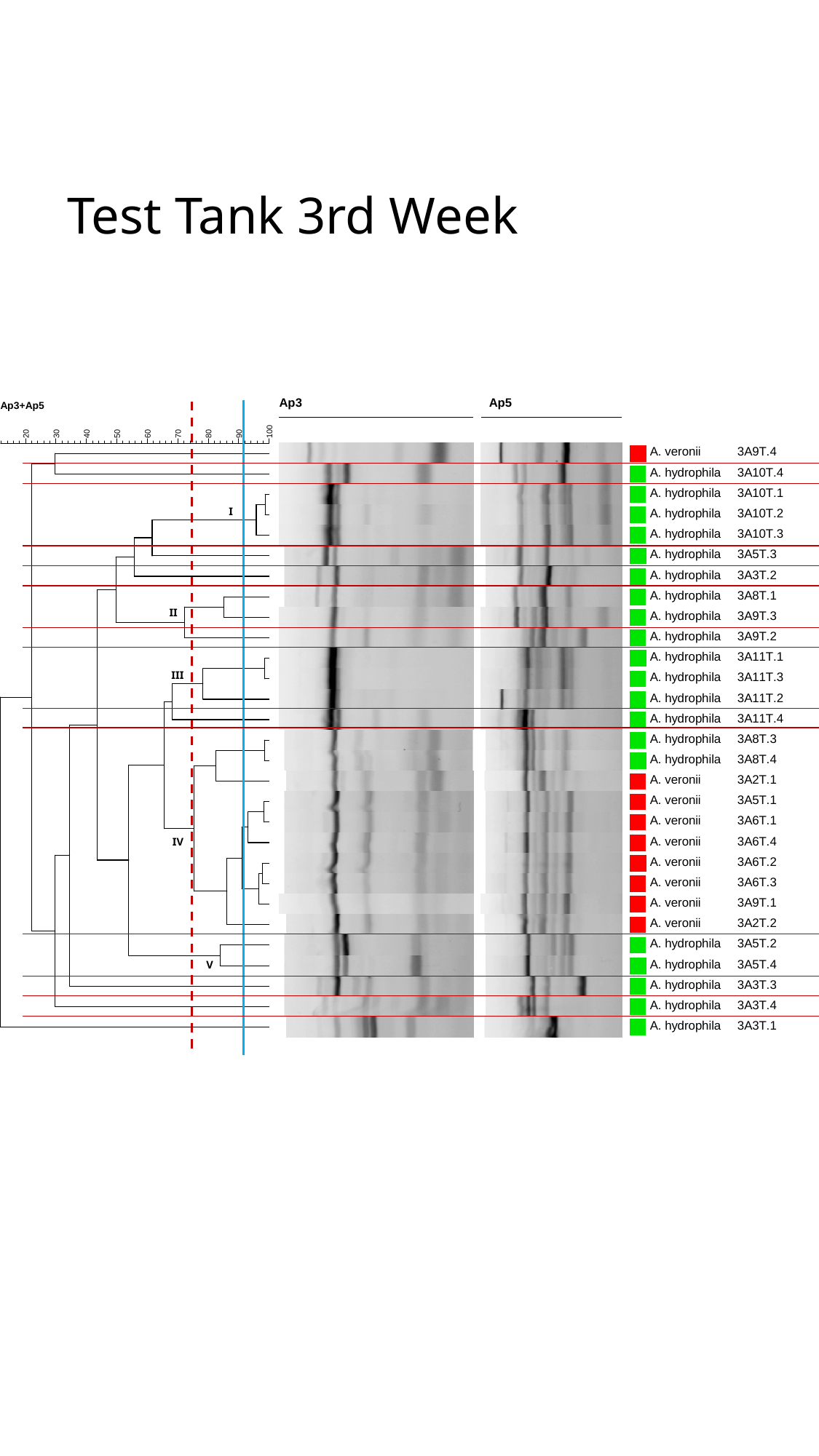

# Test Tank 3rd Week
Ap3
Ap5
I
II
III
IV
V

## Slide 9
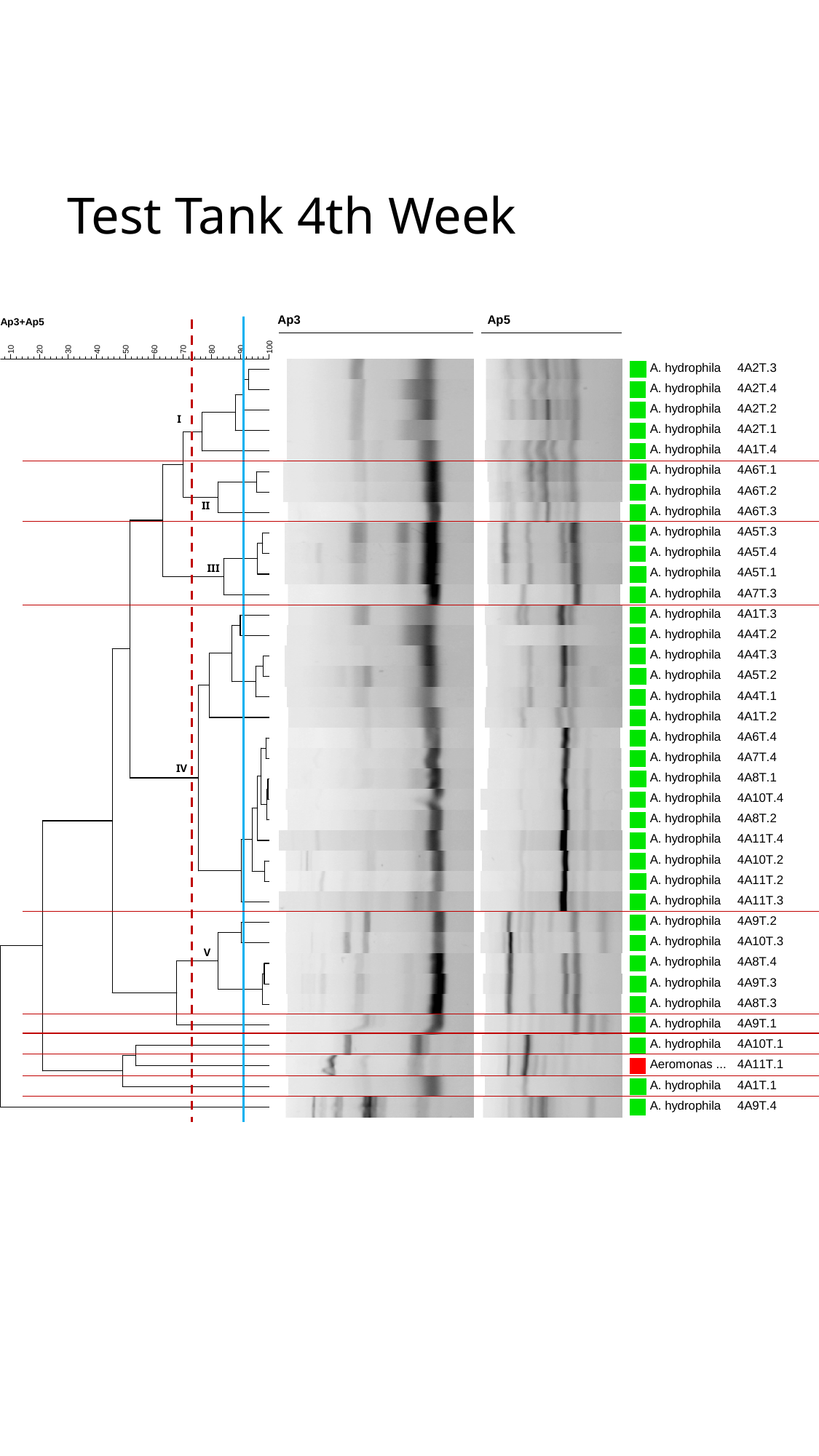

# Test Tank 4th Week
Ap3
Ap5
I
II
III
IV
V

## Slide 10
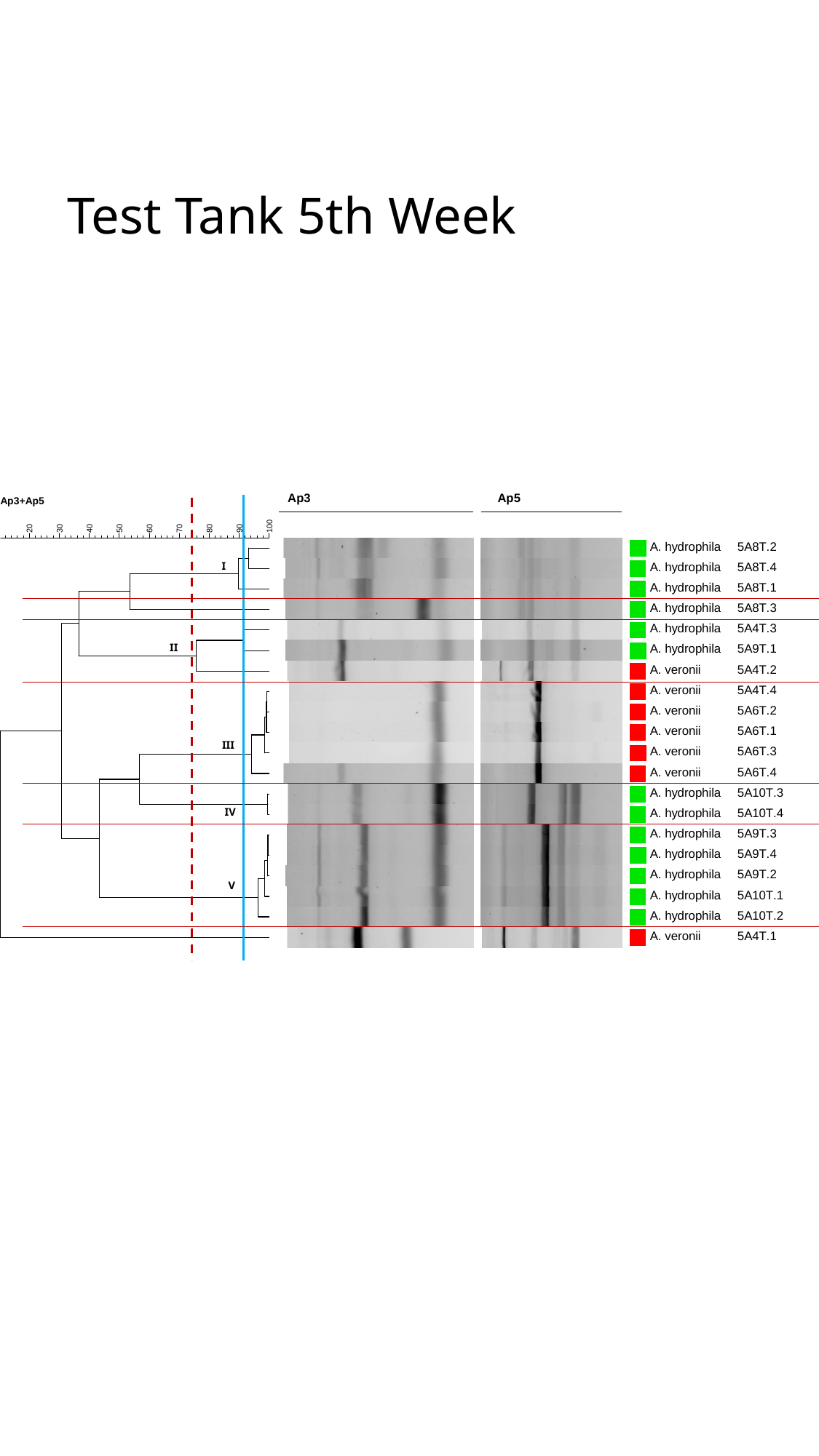

# Test Tank 5th Week
Ap3
Ap5
I
II
III
IV
V
