# Supplementary material for: Aeromonas spp. Prevalence, Virulence, and Antimicrobial Resistance in an Ex Situ Program for Threatened Freshwater Fish—A Pilot Study with Protective Measures
Source: Animals (Basel). 2022 Feb 11;12(4):436. doi: 10.3390/ani12040436 (PMC8868083; doi:10.3390/ani12040436)
Supplement: Supplementary file 1 [file animals-12-00436-s001.zip › 2.23 animals-1542936-supplementary/Supplementary Table S1.pdf]

Table 2. Operation scheme of the assay.

Week	Monday	Tuesday	Wednesday	Thursday	Friday	Saturday	Sunday
1st	Capture + Transport	Fasting	Feeding	Water Renovation	Feeding	Fasting	Fasting
	1st Bacterial Sampling		Food Sampling	Water Sampling			
2nd	Feeding	2nd Bacterial Sampling	Feeding	Water Renovation	Feeding	Fasting	Fasting
			Food Sampling	Water Sampling			
3rd	Feeding	3rd Bacterial Sampling	Feeding	Water Renovation	Feeding	Fasting	Fasting
			Food Sampling	Water Sampling			
4th	Feeding	4th Bacterial Sampling	Feeding	Water Renovation	Feeding	Fasting	Fasting
			Food Sampling	Water Sampling			
5th	Feeding	5th Bacterial Sampling					
