# Supplementary figures and images for: Aeromonas spp. Prevalence, Virulence, and Antimicrobial Resistance in an Ex Situ Program for Threatened Freshwater Fish—A Pilot Study with Protective Measures
Source: Animals (Basel). 2022 Feb 11;12(4):436. doi: 10.3390/ani12040436 (PMC8868083; doi:10.3390/ani12040436)

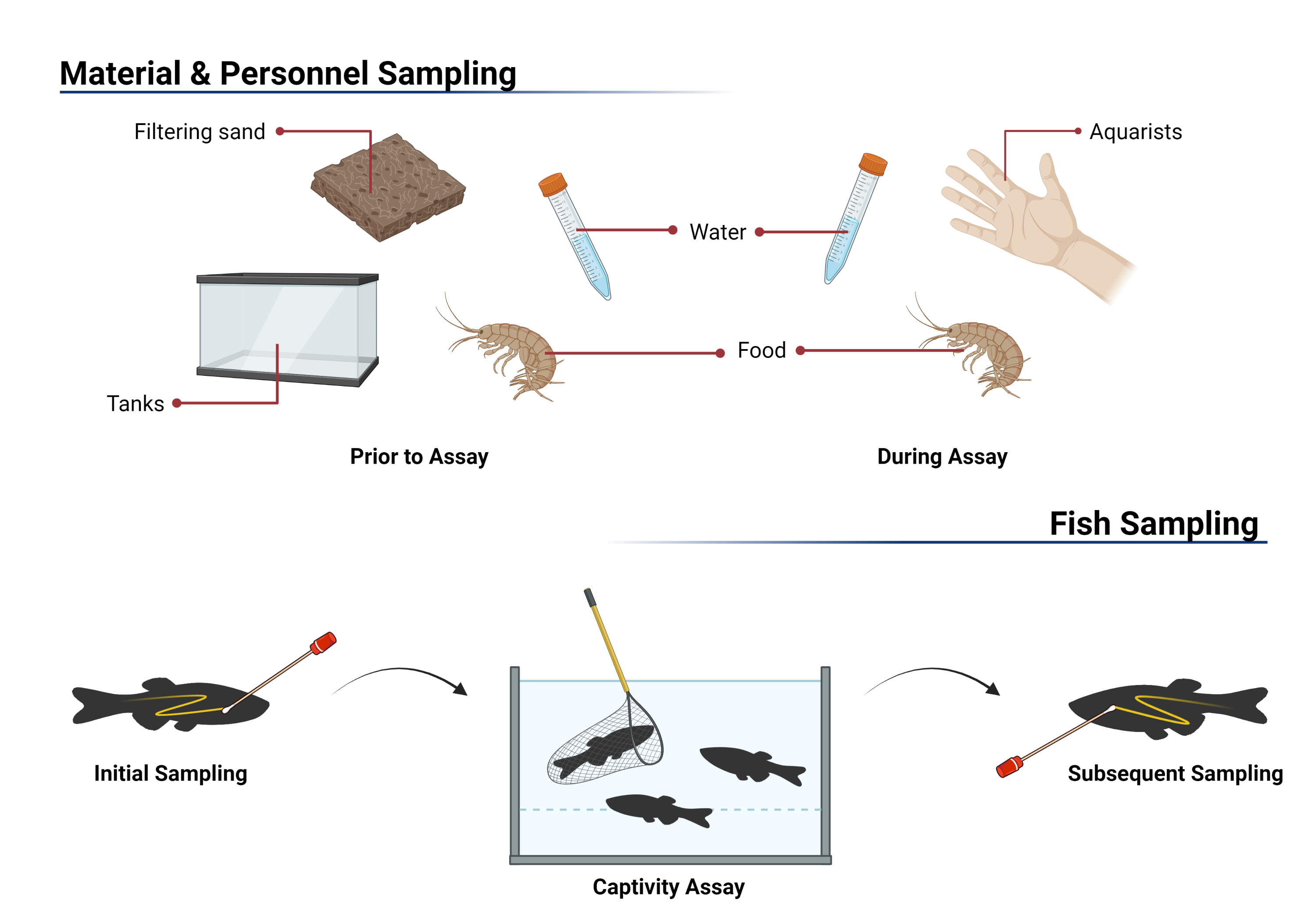

Supplement: Supplementary file 1 [file animals-12-00436-s001.zip › 2.23 animals-1542936-supplementary/Supplementary Figure S1.png]
